# Supplementary material for: Regulation of glypican 6-mediated Wnt activation maintains TDP-43 nuclear localization in neurons
Source: Sci Rep. 2025 Dec 11;16:2283. doi: 10.1038/s41598-025-32069-9 (PMC12816070; doi:10.1038/s41598-025-32069-9)

Regulation of Glypican 6-mediated Wnt activation maintains TDP-43 nuclear localization in neurons

Supplementary Information

Nan Zhang[1,2] and Shanthini Sockanathan[1*]

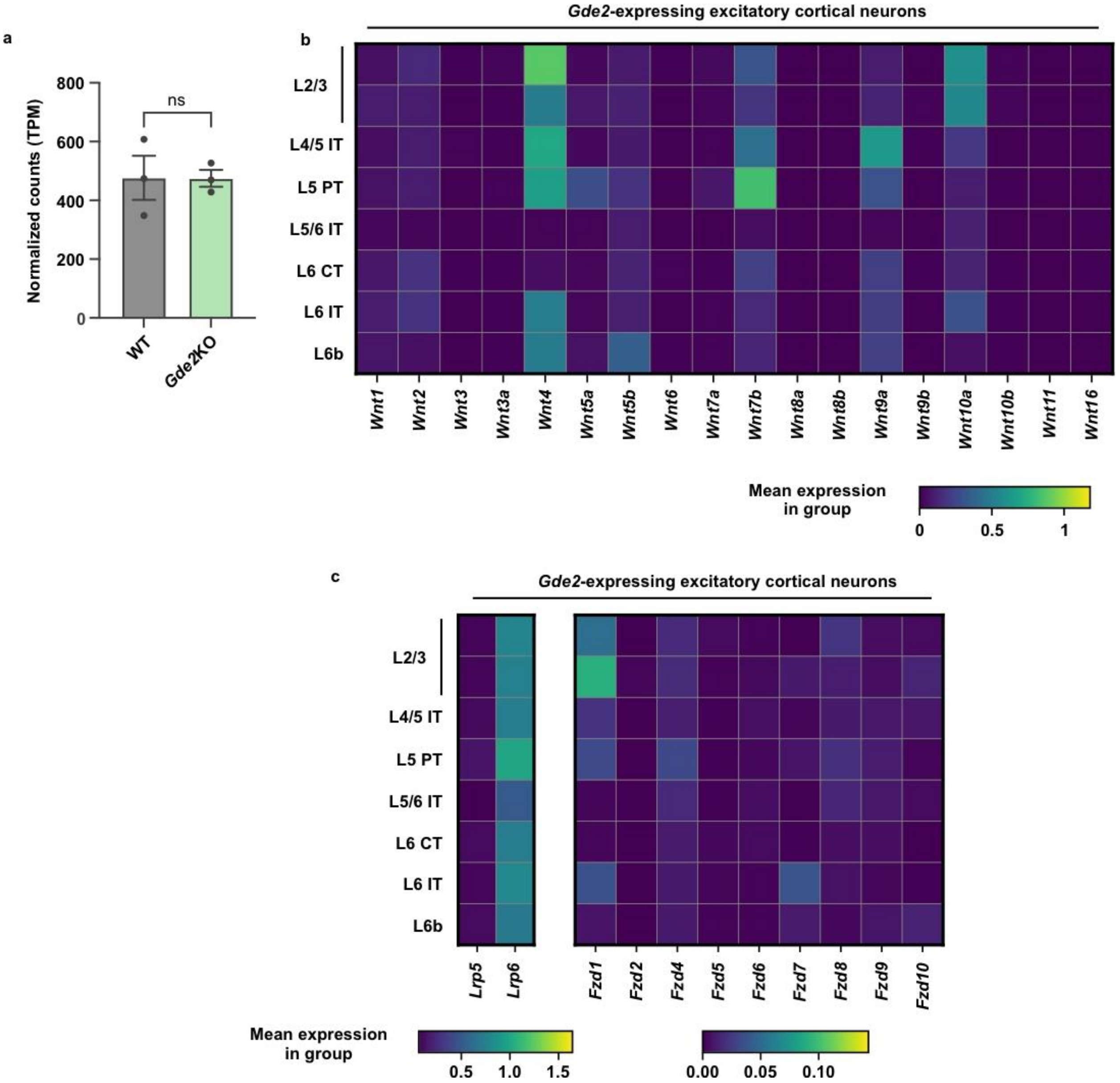

Figure S1. The expression of Wnt signaling components in *Gde2*-expressing cortical neurons.

a. Normalized transcript count in transcripts per million (TPM) of *Gpc6* in 4-month WT and *Gde2*KO cortices. Unpaired t-test, ns p = 0.9849. Data available at GEO using the accession ID GSE246462. mean $\pm$ sem, n = 3. **b-c**. Heat maps showing the expression of Wnt ligand-encoding genes, *Lrp5* and *Lrp6*, and *Fzd* family genes in *Gde2*-expressing neurons in the mouse cortex.

Each subpanel is scaled differently to highlight the range of expression levels. Data from the Allen Brain Atlas. L: layer.

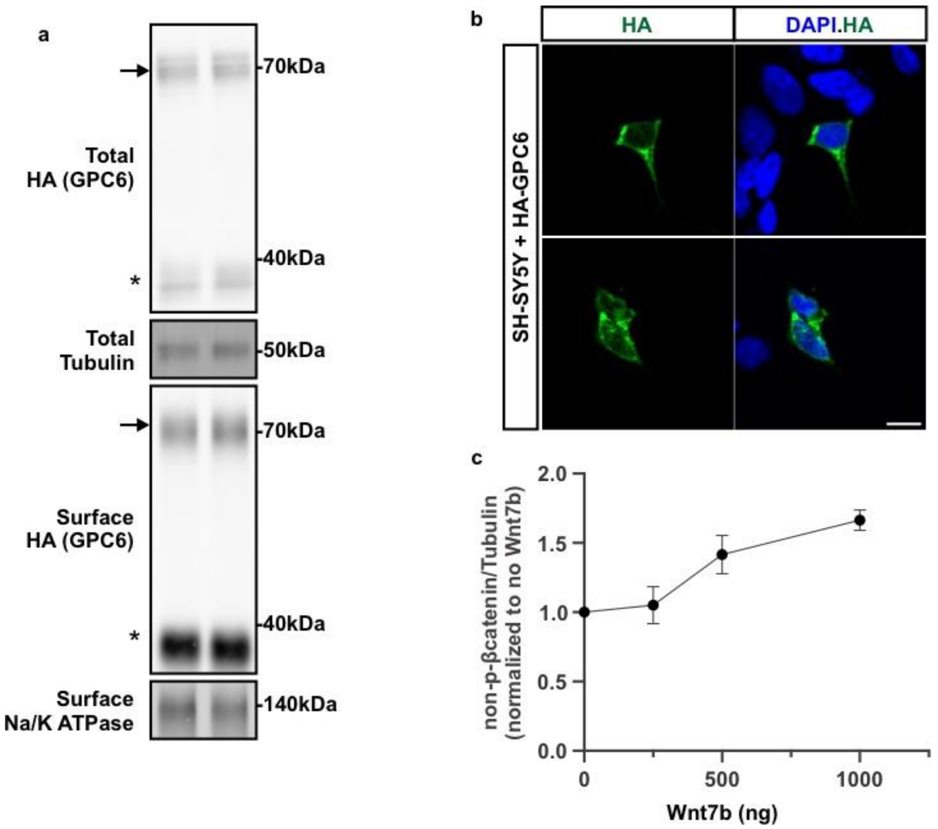

Figure S2. Validation of HA-GPC6 and Wnt7b expression in SH-SY5Y cells.

a. Representative western blot images of surface biotinylation of SH-SY5Y cells overexpressing HA-GPC6 demonstrating that HA-GPC6 is expressed at the expected molecular weight and localized on the cell surface. The arrow marks the full-length form of HA-GPC6; asterisk marks HA-GPC6 after furin cleavage. **b**. Representative images of immunostained SH-SY5Y cells showing the localization of HA-GPC6. Scale bar = 10μm. **c**. Graph quantifying non-p-β-catenin levels normalized to Tubulin in SH-SY5Y cells transfected with 0, 250, 500, and 1000ng of Wnt7b plasmid. n = 2-3, mean ± s.e.m.. Transfection of 250ng Wnt7b plasmids was used to model suboptimal Wnt7b signaling (for Fig. 2a-b) and transfection of 500ng Wnt7b plasmids was used to stimulate Wnt7b signaling (for Fig. 2c-e).

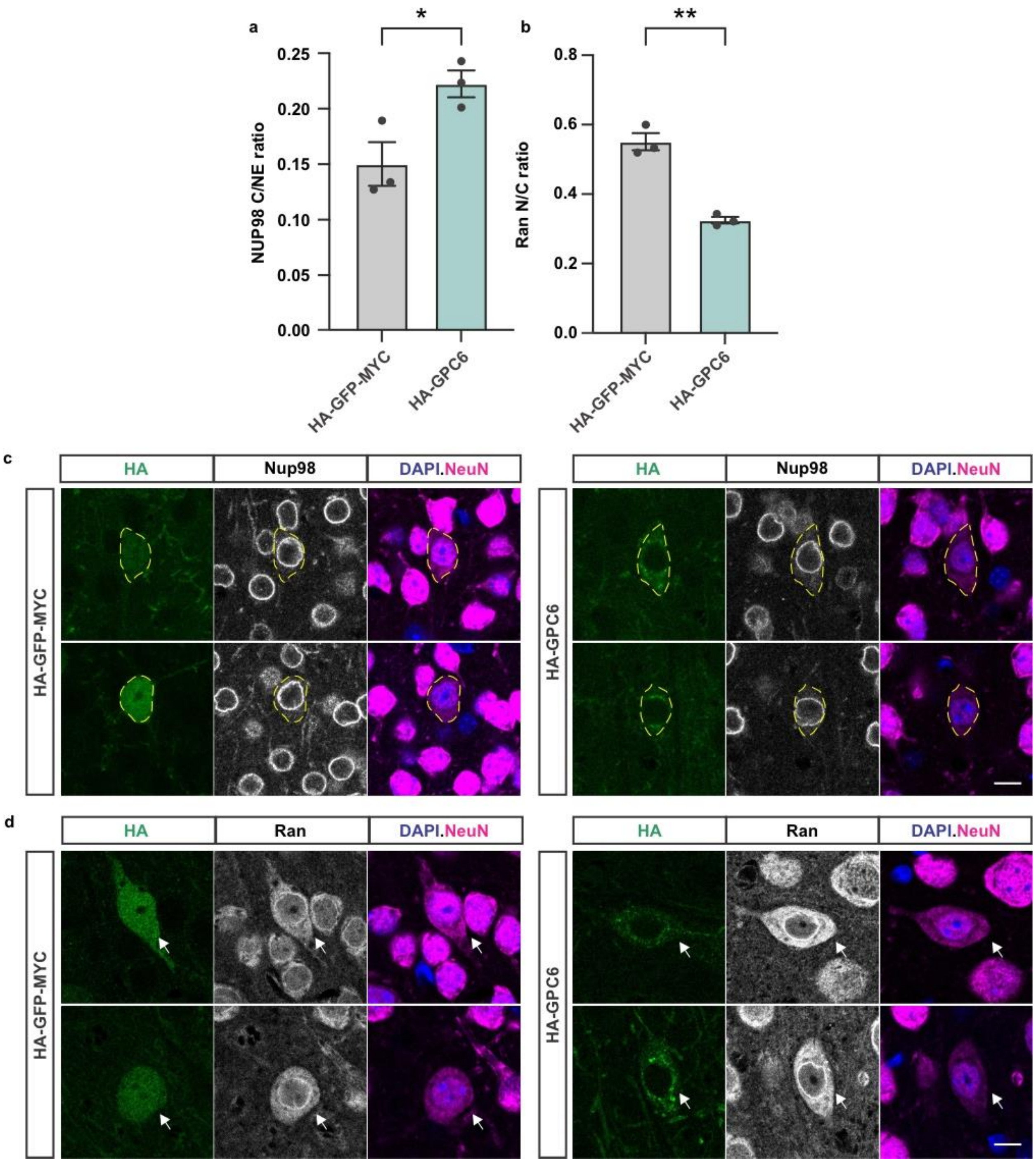

Figure S3. GPC6 overexpression activates Wnt signaling and drives NPC and NCT deficits

a-b. Graphs quantifying neurons expressing HA-GFP-MYC or HA-GPC6 comparing by animal (**a**) Nup98 C/Nuclear envelope (NE) ratio (*p = 0.0202, paired t-test. N = 3 animals, n = 78 HA-GFP-MYC, 77 HA-GPC6-expressing cells), (**b**) Ran N/C ratio (**p = 0.0057, paired t-test. N = 3 animals,

n = 79 HA-GFP-MYC, 76 HA-GPC6-expressing cells). All graphs: mean ± sem. **c-d.** Representative images of immunostained cortical sections of AAV-transduced WT;*Wnt-GFP-MYC* animals 10 days post-injection. Dashed lines outline the cell body of transduced cells in **c**; arrows highlight transduced cells in **d**. Scale bar = 10μm.

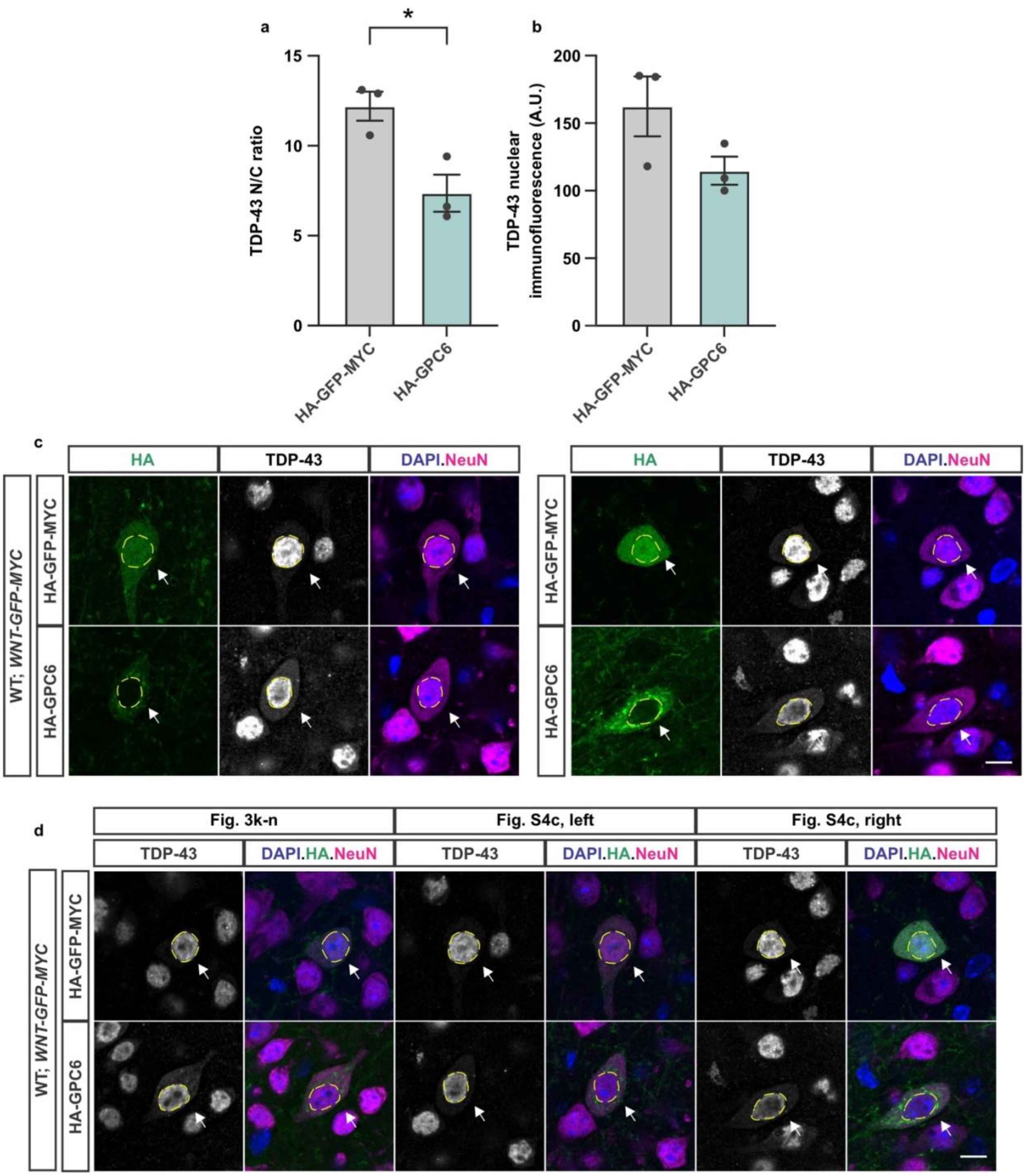

Figure S4. GPC6 overexpression drives TDP-43 abnormalities

a-b. Graphs quantifying neurons expressing HA-GFP-MYC or HA-GPC6 comparing by animal (**a**) TDP-43 N/C ratio (*p = 0.0405, paired t-test. N = 3 animals, n = 67 HA-GFP-MYC, 62 HA-GPC6-expressing cells.), (**b**) TDP-43 nuclear intensity (ns p = 0.1041, paired t-test. N = 3 animals, n =

67 HA-GFP-MYC, 62 HA-GPC6-expressing cells.). A.U.: arbitrary unit. All graphs: mean ± sem. **c.** Additional representative images of immunostained cortical sections of AAV-transduced WT;*Wnt-GFP-MYC* animals 10 days post-injection. The same brightness and contrast adjustments were applied to AAV HA-GFP-MYC and AAV HA-GPC6-MYC transduced cells and are identical to parameters used in Fig. 3k-n. d. Raw immunostaining images of Fig. 3k-n and **Fig. S4c** without brightness and contrast adjustment. Arrows highlight transduced cells, and dashed lines highlight the nuclei of transduced cells. Scale bar = 10μm.

Uncropped immunoblot images

Figure 1d:

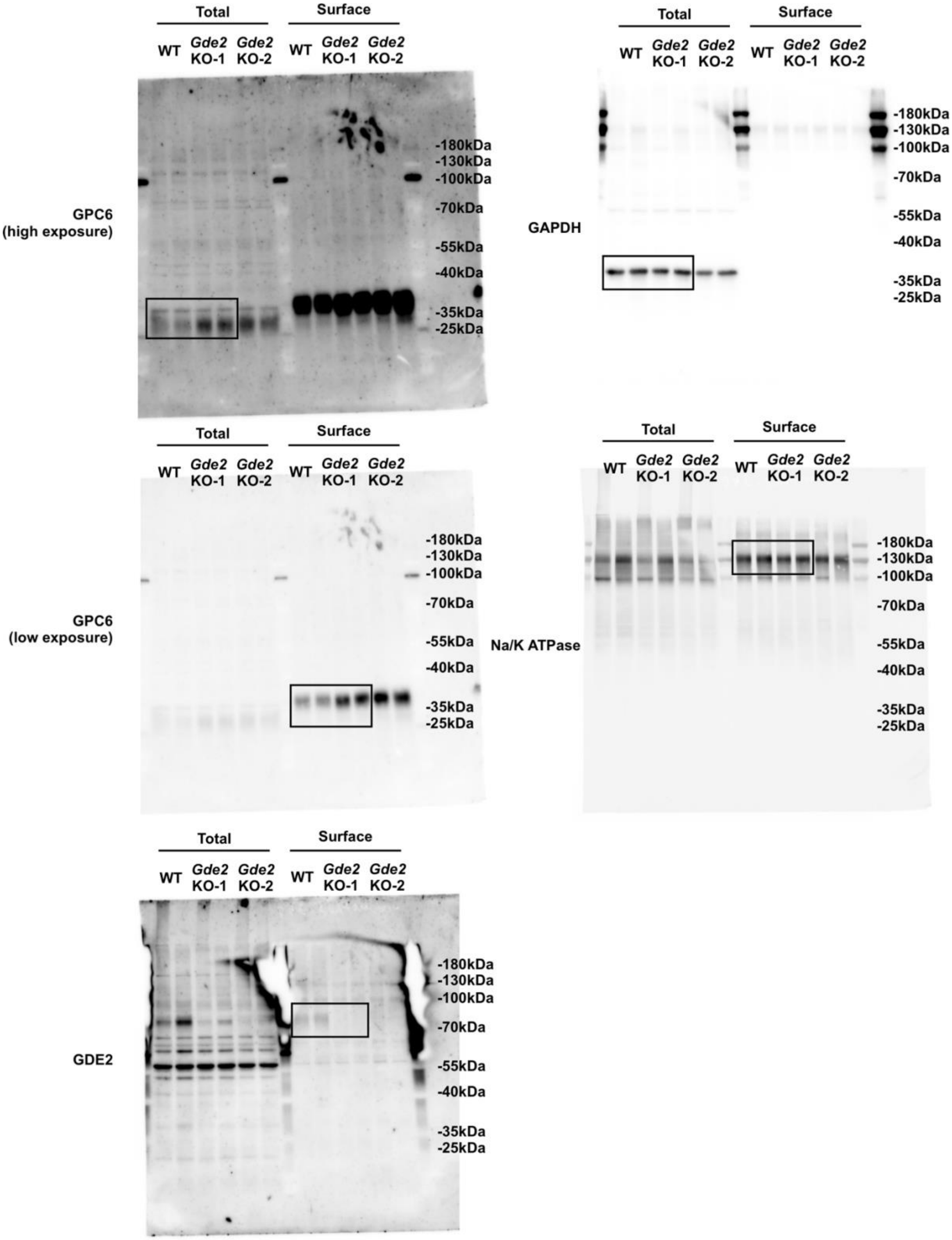

Figure 2a:

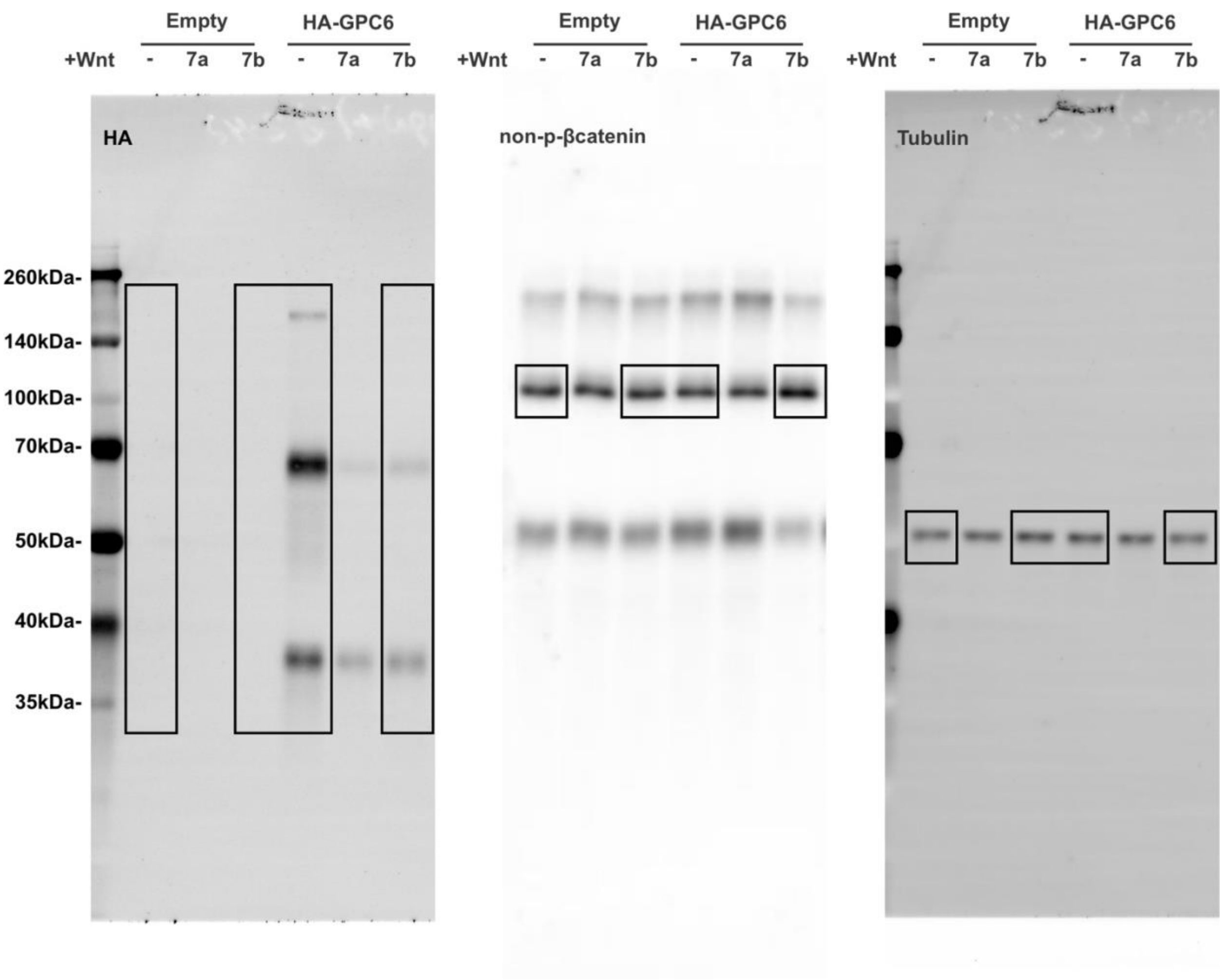
Empty
HA-GPC6
+Wnt
-
7a
7b
-
7a
7b
HA
non-p-βcatenin
Tubulin
260kDa-
140kDa-
100kDa-
70kDa-
50kDa-
40kDa-
35kDa-

Figure 2d:

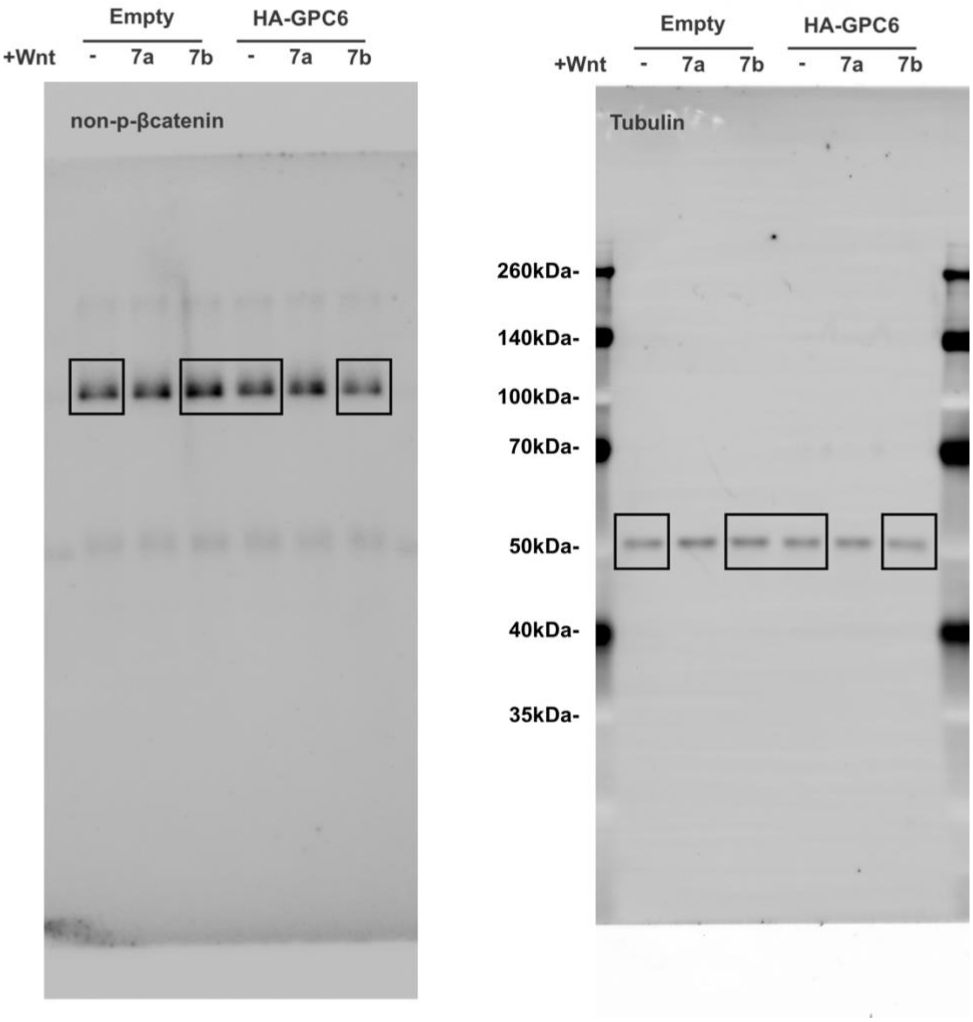
Empty
HA-GPC6
+Wnt
-
7a
7b
-
7a
7b
non-p-βcatenin
Empty
HA-GPC6
+Wnt
-
7a
7b
-
7a
7b
Tubulin
260kDa-
140kDa-
100kDa-
70kDa-
50kDa-
40kDa-
35kDa-

Figure 4a:

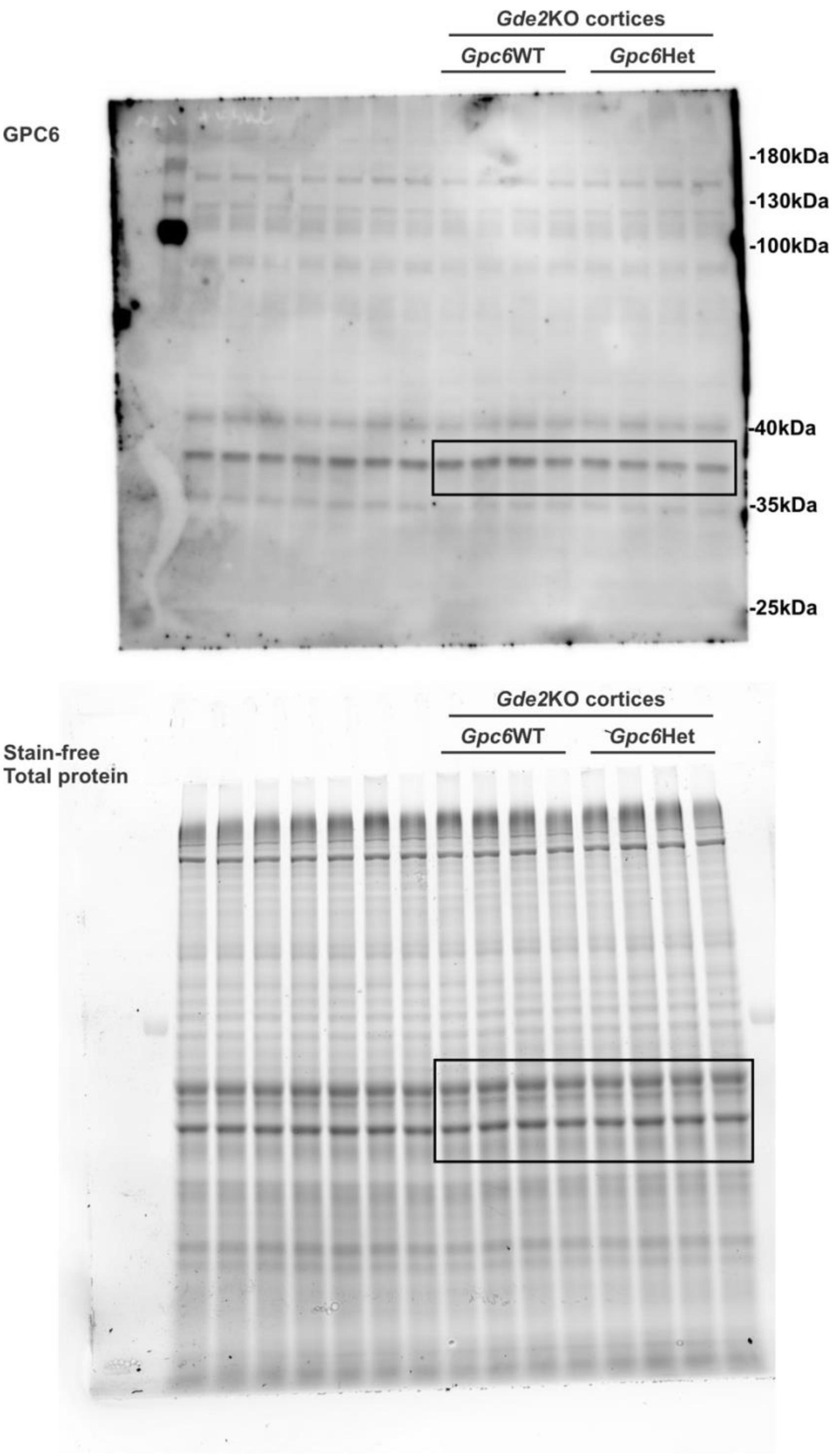
Gde2KO cortices
Gpc6WT
Gpc6Het
GPC6
-180kDa
-130kDa
-100kDa
-40kDa
-35kDa
-25kDa
Gde2KO cortices
Gpc6WT
Gpc6Het
Stain-free
Total protein

Figure S2:

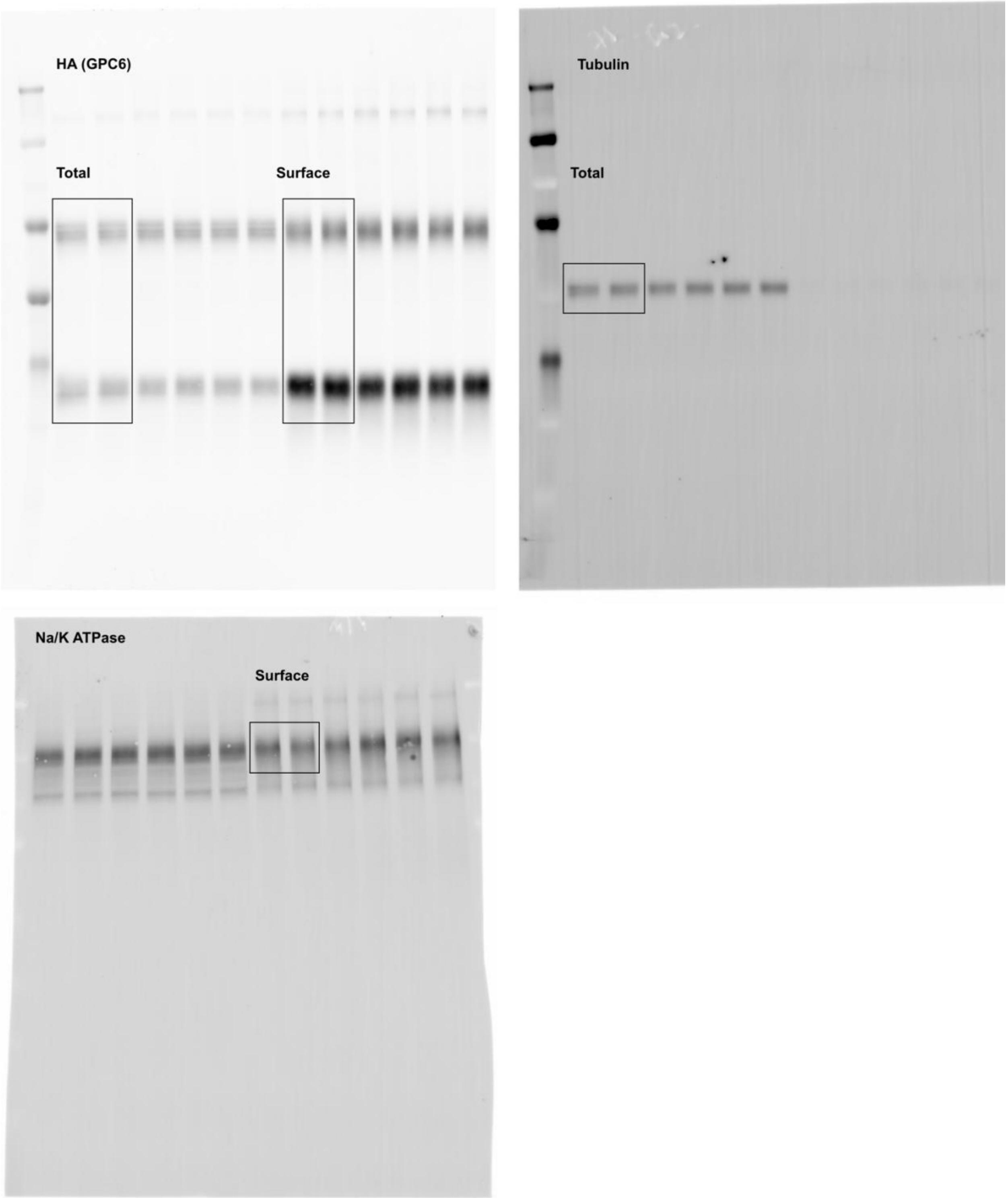

Supplement: Supplementary file 1 — Supplementary Material 1 [file 41598_2025_32069_MOESM1_ESM.pdf]
